# Successful outcome achieved with adjuvant chemotherapy with irinotecan plus cisplatin in rectal neuroendocrine carcinoma: a case report

**DOI:** 10.1186/s40792-024-02010-9

**Published:** 2024-09-19

**Authors:** Yoshitaka Saegusa, Shintaro Akabane, Manabu Shimomura, Hiroshi Okuda, Takuya Yano, Tetsuya Mochizuki, Wako Inoue, Mizuki Yamaguchi, Shinji Yamaguchi, Kazuhiro Sentani, Masami Yamauchi, Kentaro Tokumo, Hideki Ohdan

**Affiliations:** 1 Department of Gastroenterological and Transplant Surgery, Graduate School of Biomedical and Health Science, Hiroshima University, Hiroshima, Japan; 2 Department of Molecular Pathology, Graduate School of Biomedical and Health Science, Hiroshima University, Hiroshima, Japan; 3 Department of Clinical Oncology, Hiroshima University Hospital, Hiroshima, Japan

**Keywords:** Neuroendocrine carcinomas, Adjuvant chemotherapy, Irinotecan, Cisplatin, Case report

## Abstract

**Background:**

Rectal neuroendocrine carcinomas (NECs) are rare and associated with poorer prognoses compared to conventional adenocarcinomas. The efficacy of adjuvant chemotherapy for resectable rectal NECs remains uncertain. Herein, we present a case of rectal NEC successfully treated with postoperative chemotherapy using irinotecan plus cisplatin.

**Case presentation:**

A 48-year-old woman with a history of endometrial cancer presented with an intramural rectal tumour detected on follow-up imaging. Colonoscopy revealed a 30 mm submucosal tumour, and laparoscopic low anterior resection was performed. Histopathological examination showed poorly differentiated atypical cells with solid growth patterns. Metastasis from the uterine cancer was ruled out due to histological differences between the primary uterine tumour and the rectal lesion, as well as the absence of hormone receptor immunohistochemical expression. Further immunohistochemical analysis revealed diffuse CD56 positivity, a high mitotic rate (> 20/10 high power fields) and a Ki-67 labelling index exceeding 70%. Based on these findings, a diagnosis of rectal NEC, T3N0M0, Stage IIB (UICC 8th edition), was established. Given the aggressive nature of the tumour evidenced by a high Ki-67 labelling index, adjuvant chemotherapy comprising six cycles of irinotecan plus cisplatin was administered to mitigate the risk of recurrence. At the 3-year follow-up, the patient was free of disease recurrence.

**Conclusion:**

This case highlights the importance of multidisciplinary surgical interventions followed by adjuvant chemotherapy in managing rectal NECs.

## Background

Rectal neuroendocrine carcinomas (NECs) are rare tumours with more aggressive behaviour and poorer prognosis than conventional adenocarcinomas [1]. Several studies have demonstrated the efficacy of cisplatin-based chemotherapy for advanced gastrointestinal NECs [2]. For the treatment of resectable rectal NECs, surgery alone is not preferable, and adjuvant chemotherapy needs to be employed considering the potential malignancy. However, no regimens have been established to date. Herein, we describe a case of rectal NEC treated with laparoscopic resection and sequential chemotherapy with irinotecan plus cisplatin (IP).

## Case presentation

**Fig. 1:**
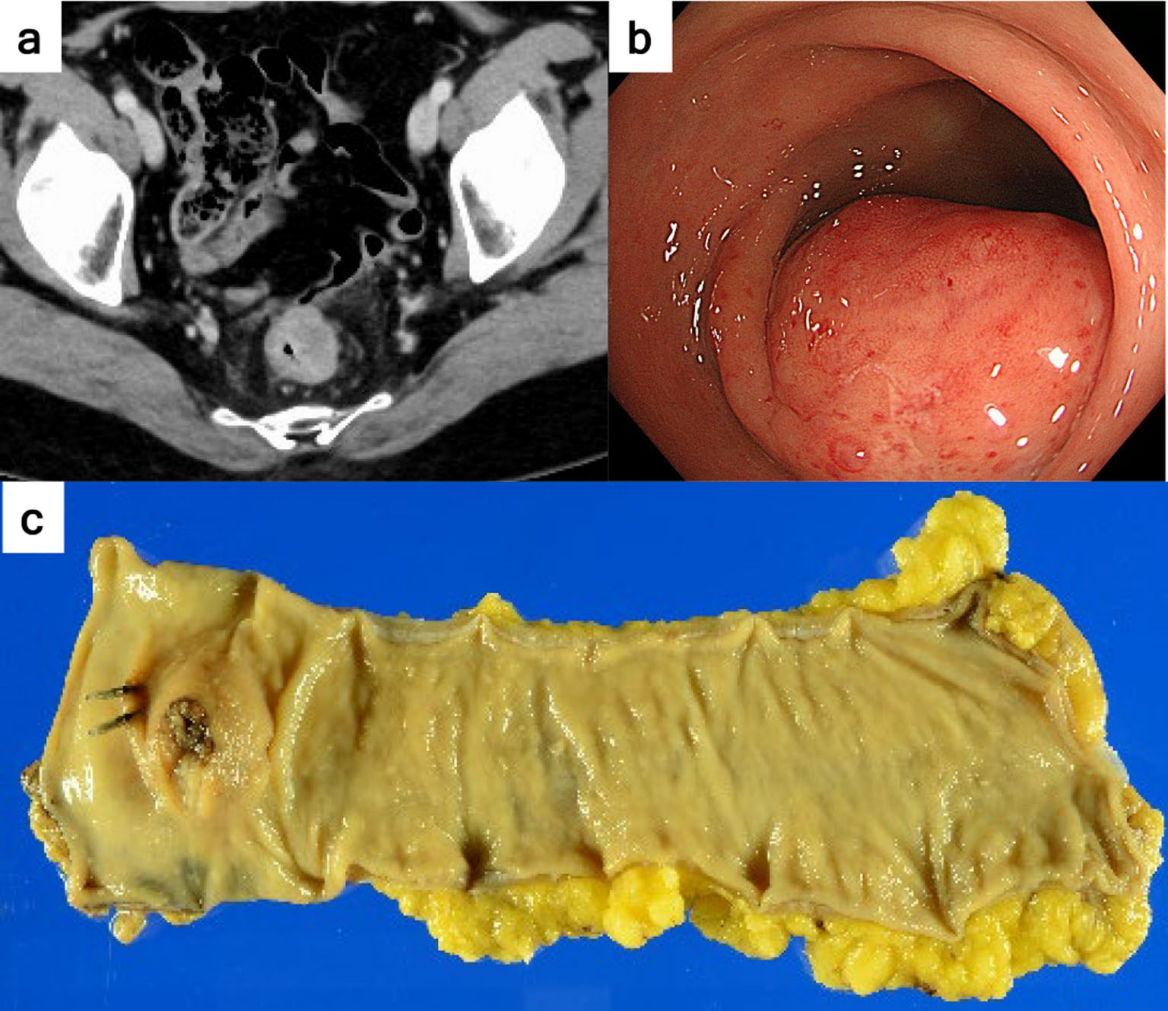
Images of the rectal tumour. **a** CT tomography revealed an intramural tumour in the rectum. **b** Colonoscopy reveals a submucosal tumour. **c** Resected specimen showing a submucosal tumour with ulceration

A 48-year-old woman with a history of total hysterectomy and bilateral salpingo-oophorectomy for stage IA endometrial cancer (UICC 8th edition) showed an intramural tumour in the rectum on follow-up CT scan (Fig. 1a). Colonoscopy revealed a 30 mm-sized submucosal tumour (Fig. 1b), and a subsequent biopsy showed a moderately differentiated adenocarcinoma. Magnetic resonance imaging (MRI) and positron emission tomography–computed tomography (PET–CT) scans were also conducted (images not shown), estimating the preoperative stage as cT3N0M0, corresponding to stage IIA (UICC 8th edition). We performed laparoscopic low anterior resection with D3 lymph node dissection, and the surgical specimen showed a submucosal tumour with ulceration 3.5 × 2.5 cm in size (Fig. 1c). The patient was discharged without major complications 9 days postoperatively.

**Fig. 2:**
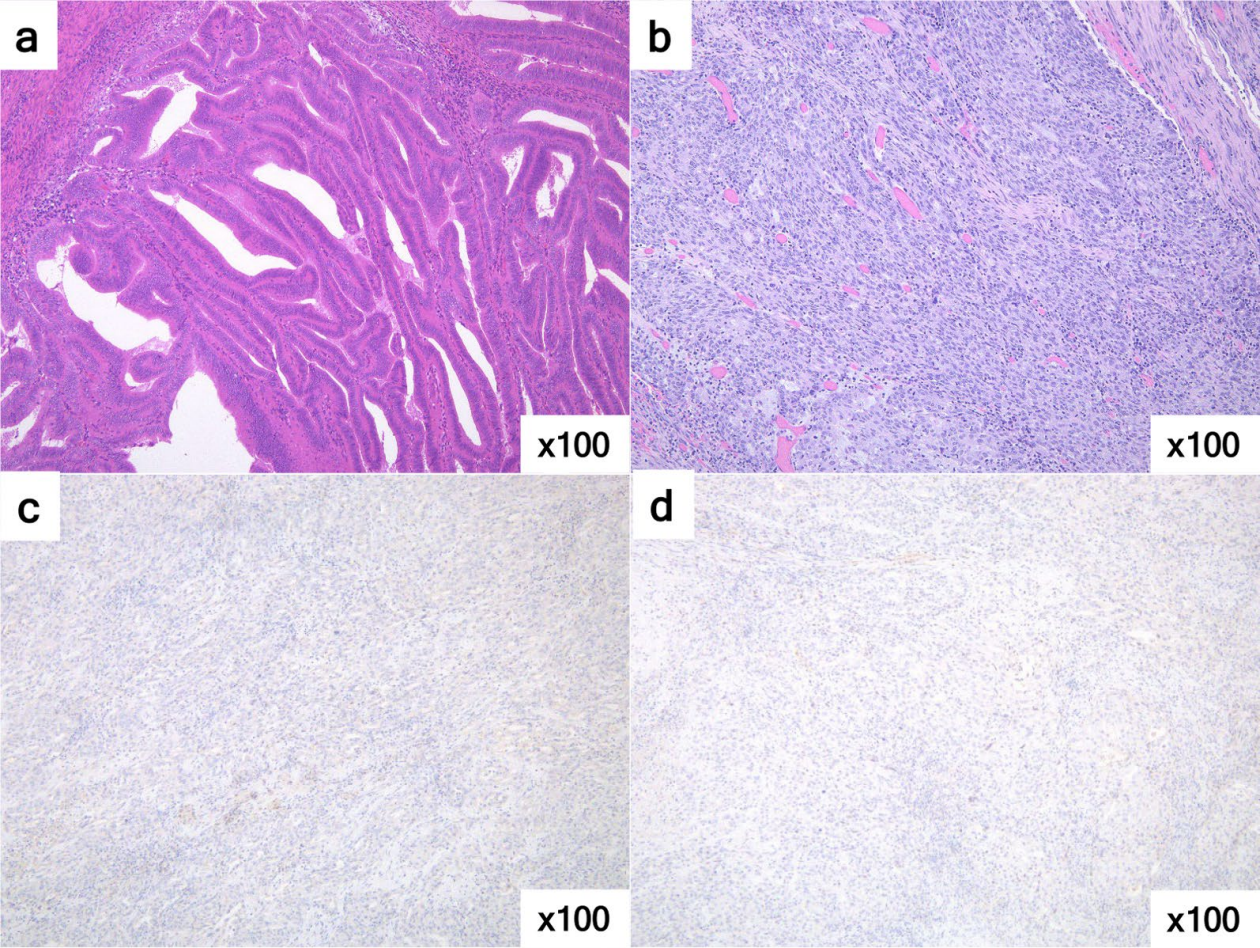
Pathological findings differentiating from endometrial cancer. **a** Well-differentiated endometrioid adenocarcinoma limited to the endometrium (H&E staining ×100). **b** Solidary proliferation of atypical cells was observed in the rectal tumour (H&E staining ×100). Carcinoma cells were negative for oestrogen receptor (**c**) and progesterone receptor (**d**) (×100)

**Fig. 3:**
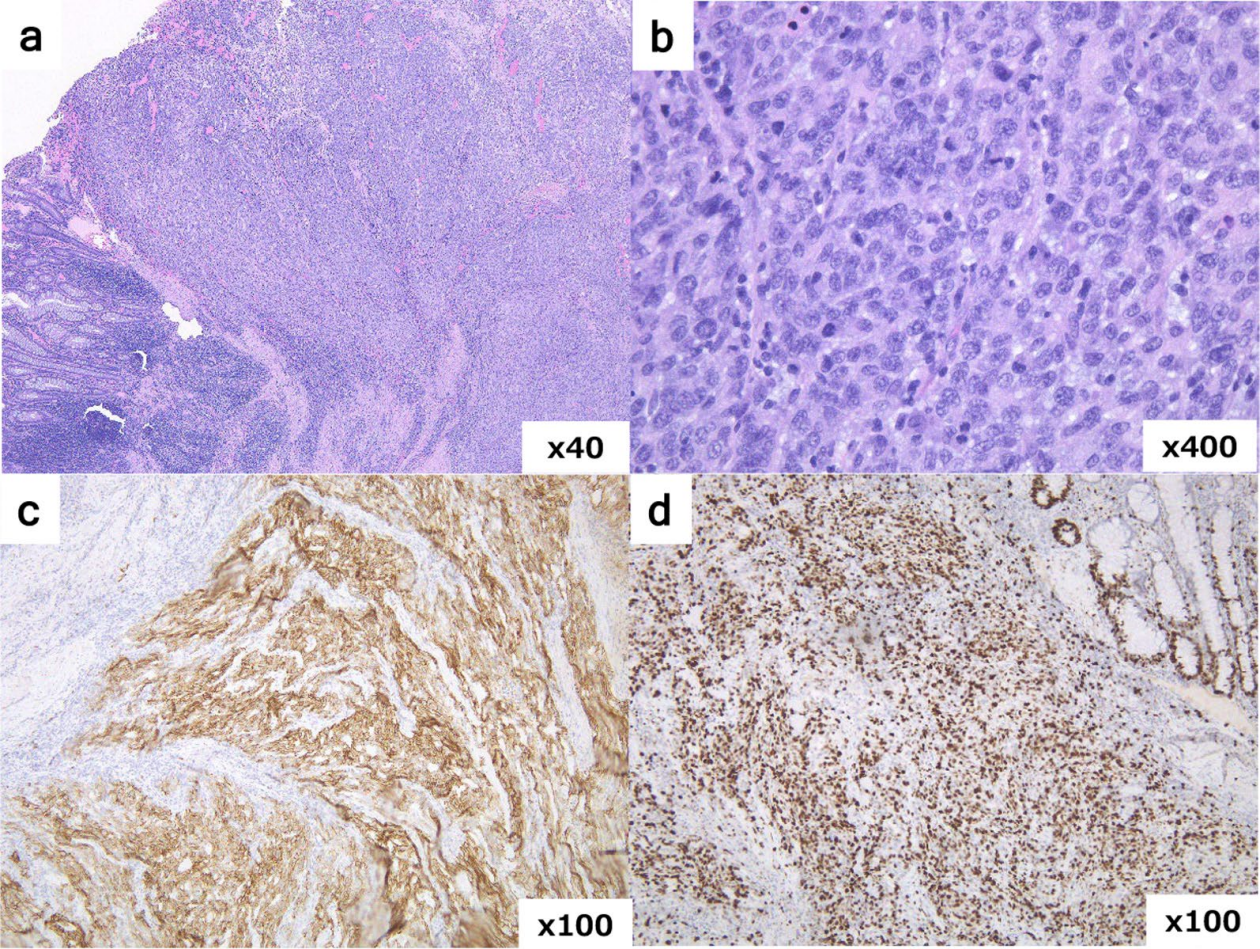
Pathological diagnosis for rectal neuroendocrine carcinoma. Microscopic findings of the rectal tumour (**a** H&E staining ×40, **b** H&E staining ×400). **c** Carcinoma cells are diffuse positive for CD56 (on immunohistochemistry, ×100). **d** Ki-67 labelling index > 70% in tumour cells (on immunohistochemistry, ×100)

Histopathological examination revealed poorly differentiated, proliferating, atypical cells with solid growth patterns. A metastatic tumour from the uterine cancer was deemed unlikely because of histological discrepancies between the primary uterine tumour and the rectal lesion, as well as the absence of oestrogen and progesterone receptor expression (Fig. 2). Immunohistochemical analysis revealed positivity for CK7 and negativity for CK20 and CDX2 (data not shown). The cytokeratin staining pattern did not indicate conventional rectal adenocarcinoma, and microsatellite unstable rectal cancer was suspected considering the poorly differentiated histology [3]. As this patient met the criteria of the Bethesda Guidelines 2004, we also needed to exclude the possibility of Lynch syndrome. We then performed immunostaining for mismatch repair (MMR) proteins and found that the MMR protein expression was intact (data not shown), indicating that the tumour developed due to factors other than microsatellite instability [4]. Further immunohistochemical analysis demonstrated diffuse CD56 positive expression, negative for synaptophysin and chromogranin A. Mitotic activity was more than 20/10 high power fields and the Ki-67 labelling index was more than 70% in the tumour cells (Fig. 3). From these findings, we diagnosed neuroendocrine carcinoma of the rectum pT3N0M0, Stage IIB (UICC 8th edition). Although there were no lymph node metastases, the risk of recurrence was high because of the high Ki-67 labelling index. Consequently, we administered six courses of adjuvant chemotherapy with IP. The patient experienced G3 neutropenia, the overall adverse effects were manageable, and the treatment regimen was safely completed. The patient remained alive and recurrence free at the follow-up of 3 years.

## Discussion

This case report describes the favourable clinical course of a patient with rectal NEC who underwent surgical resection and subsequent adjuvant chemotherapy. Evidence of treatment strategies for rectal NEC is lacking because of the extremely low incidence of this disease [5]. This report supports accumulating evidence for the combination of radical resection and systemic chemotherapy as a treatment strategy for rectal NEC.

Neuroendocrine neoplasms (NENs) were formerly known as carcinoids; however, the understanding of the disease is ambiguous. However, as pathological investigations progressed, the recognition of diverse grades led to the proposal of the 2010 WHO Classification [6]. NENs with poorly differentiated histology and a Ki-67 index > 20% were designated as neuroendocrine carcinomas. Gastrointestinal NECs exhibit a poor prognosis owing to their biological malignancy and propensity for metastasis, showing morphological features similar to those of small cell carcinomas of the lung [7]. In this case, biopsy tissue at the time of preoperative endoscopy showed that the tumour tissue was consistent with a moderately differentiated adenocarcinoma, and there were no findings that would raise active suspicion of NEC. The difficulty in preoperative diagnosis may be attributed to the submucosal localisation of the tumour, and previous report suggests that the positive diagnosis rate is as low as 60% [1].

Bernick et al. reported that colorectal NECs have a poor prognosis, with a median survival of only 10.4 months, indicating the necessity for multidisciplinary treatment [1]. Further evidence showed that patients with a higher Ki-67 index had longer overall survival with platinum-based therapy than those with a lower Ki-67 index in advanced gastrointestinal NEC [8]. Several case reports demonstrated the efficacy of perioperative chemotherapy for colorectal NECs following surgical resection [7, 9, 10]. Large database analyses suggest that adjuvant chemotherapy extends overall survival in colorectal neuroendocrine carcinomas following curative resection, regardless of the disease stage [11]. In a retrospective analysis involving lymph node-negative cases of rectal NECs, it was demonstrated that the median overall survival was 1.0 year for surgery alone, whereas it was 2.5 years for patients treated with adjuvant chemotherapy [12]. Given the shared micromorphological features of small cell lung cancer, treatment regimens for pancreatic and gastrointestinal neuroendocrine carcinomas are influenced by those for small cell lung cancers, with cisplatin-based adjuvant chemotherapy recommended following resection [13].

There is still much debate regarding the appropriate regimens for colorectal NECs. A phase 3 randomised clinical trial for advanced neuroendocrine carcinomas of the digestive system conducted in Japan determined that both IP and cisplatin plus etoposide (EP) were suitable standard treatments, demonstrating no significant superiority over the others [2]. Nevertheless, in another clinical trial conducted for advanced small cell lung cancer, IP was superior to EP [14].

In our case, we selected the IP regimen based on these considerations and administered it post-operatively, yielding favourable outcomes. Although there are several reports of long-term survival with multidisciplinary treatment for colorectal neuroendocrine carcinomas [15, 16], issues regarding the choice of multimodal treatment remain unclear. A treatment strategy with the accrual of additional cases needs to be formulated. It is necessary to establish higher level of evidence by large-scale clinical trials for colorectal NECs, including cases without lymph node metastases.

## Conclusion

This case report describes a rectal NEC that was successfully treated with surgical resection, followed by adjuvant chemotherapy with an IP regimen. This case highlights the importance of a multidisciplinary treatment for resectable gastrointestinal NECs.

## Data Availability

Not applicable.

## Abbreviations

NEC: Neuroendocrine carcinoma
CT: Computed tomography
UICC: Union for International Cancer Control
IP: Irinotecan plus cisplatin
EP: Cisplatin plus etoposide
